# Perinatal acquisition of drug-resistant HIV-1 infection: mechanisms and long-term outcome

**DOI:** 10.1186/1742-4690-6-85

**Published:** 2009-09-19

**Authors:** Constance Delaugerre, Marie-Laure Chaix, Stephane Blanche, Josiane Warszawski, Dorine Cornet, Catherine Dollfus, Veronique Schneider, Marianne Burgard, Albert Faye, Laurent Mandelbrot, Roland Tubiana, Christine Rouzioux, the ANRS French Perinatal Cohort

**Affiliations:** 1 EA 3620 MRT, Descartes University, Paris, France; 2 Virology Department, Necker-Enfants Malades Hospital-APHP, Paris, France; 3 Hematology Immunology Peadiatric Department, Necker-Enfants Malades Hospital-APHP, Paris, France; 4 INSERM unit U822, University Paris-Sud, Le Kremlin Bicêtre, France; 5 Pediatric and Oncology Department, Trousseau Hospital-APHP, Paris, France; 6 Virology Department, Tenon Hospital-APHP, Paris, France; 7 Hematology Immunology Peadiatric Department, Robert Debre Hospital-APHP, Paris, France; 8 Gynecology Obstetric Department, Louis Mourier Hospital-APHP, Colombes, France; 9 Infectious Diseases Department, Pitie Salpetriere Hospital-APHP, Paris, France

## Abstract

**Background:**

Primary-HIV-1-infection in newborns that occurs under antiretroviral prophylaxis that is a high risk of drug-resistance acquisition. We examine the frequency and the mechanisms of resistance acquisition at the time of infection in newborns.

**Patients and Methods:**

We studied HIV-1-infected infants born between 01 January 1997 and 31 December 2004 and enrolled in the ANRS-EPF cohort. HIV-1-RNA and HIV-1-DNA samples obtained perinatally from the newborn and mother were subjected to population-based and clonal analyses of drug resistance. If positive, serial samples were obtained from the child for resistance testing.

**Results:**

Ninety-two HIV-1-infected infants were born during the study period. Samples were obtained from 32 mother-child pairs and from another 28 newborns. Drug resistance was detected in 12 newborns (20%): drug resistance to nucleoside reverse transcriptase inhibitors was seen in 10 cases, non-nucleoside reverse transcriptase inhibitors in two cases, and protease inhibitors in one case. For 9 children, the detection of the same resistance mutations in mothers' samples (6 among 10 available) and in newborn lymphocytes (6/8) suggests that the newborn was initially infected by a drug-resistant strain. Resistance variants were either transmitted from mother-to-child or selected during subsequent temporal exposure under suboptimal perinatal prophylaxis. Follow-up studies of the infants showed that the resistance pattern remained stable over time, regardless of antiretroviral therapy, suggesting the early cellular archiving of resistant viruses. The absence of resistance in the mother of the other three children (3/10) and neonatal lymphocytes (2/8) suggests that the newborns were infected by a wild-type strain without long-term persistence of resistance when suboptimal prophylaxis was stopped.

**Conclusion:**

This study confirms the importance of early resistance genotyping of HIV-1-infected newborns. In most cases (75%), drug resistance was archived in the cellular reservoir and persisted during infancy, with or without antiretroviral treatment. This finding stresses the need for effective antiretroviral treatment of pregnant women.

## Background

Mother-to-child transmission (MTCT) of HIV-1 mainly occurs during the third trimester of pregnancy or at delivery, in the absence of breastfeeding [1]. Transmission can be prevented by treating the pregnant woman during the third trimester and at delivery, and by giving the child prophylactic treatment during the first weeks of life. The efficacy of this approach was first demonstrated in 1994 with zidovudine [2], and the transmission rate has gradually fallen in Europe and the United States from 25% to below 2% [3,4]. French guidelines published in 2004 recommend starting combination antiretroviral therapy (HAART) at the end of the second trimester and adding intravenous zidovudine (ZDV) during labor. Infants receive ZDV orally for 6 weeks, alone or combined with other antiretroviral drugs if the risk of transmission is high [5].

Situations of particular risk of HIV-1 MTCT [4] include unknown maternal HIV-1 serostatus; ineffective maternal ART; maternal primary HIV-1 infection during pregnancy; and suboptimal MTCT prevention.

Infants may be at an increased risk of infection by drug-resistant HIV-1 strains when the mother harbors such viruses or when drug pressure during MTCT prophylaxis is suboptimal.

Vertical transmission of drug-resistant HIV-1 was first reported sporadically [6-8], but it is now known that 9% to 30% of infected infants exposed to MTCT prophylaxis with ZDV acquire ZDV-resistant viruses [7,9-12]. Masquelier *et al*. reported finding viruses with ZDV genotypic resistance in 20% of 34 HIV-1-infected infants who were born in France between 1994 and 1996 and were enrolled in the ANRS-EPF French national cohort [7]. In New York State, drug resistance mutations were detected in 12% of perinatally infected infants born in 1998 and 1999 [13] and in 19.1% of such infants born in 2001 and 2002 [14].

In France, between 1997 and 2004, the estimated MTCT rate was 1.8% (92 newborns). Here we report the current rate of HIV-1 drug resistance in French neonates born to infected mothers. We also report our investigation as to how these resistant viruses were acquired by the newborns, and the outcome of resistance during infancy.

## Patients and methods

### Study population

Since 1985, the ANRS French Perinatal Cohort (CO 01-ANRS-EPF, *Agence Nationale de Recherche sur le SIDA-Enquête Périnatale Française*) has prospectively collected data on HIV-infected pregnant women and their children in 90 centers throughout France. Informed consent is obtained from the mothers during pregnancy or at the time of delivery. The children receive standard care, including clinical and biological examinations at birth and 1, 3, 6, 12 and 18-24 months, as previously reported [15]. The cohort study was approved by the Cochin Hospital Institutional Review Board and by the French computer database watchdog commission (CNIL). Mother and infant plasma and cells were collected between 1990 and 2005 and stored in Necker hospital virology laboratory.

HIV-1 infection was diagnosed in the newborn when at least two separate samples were positive by HIV-1 RNA/DNA detection or by a viral culture. A positive test at birth or before 7 days of age indicates intrauterine transmission, while a negative test at birth and a positive test more than 7 days later indicate *intrapartum *transmission. An infant is considered uninfected when two tests performed one month after discontinuation of antiretroviral prophylaxis are negative.

Newborns were included in this analysis if: (1) they were born and enrolled in metropolitan France in centers participating in the EPF cohort between 1997 and 2004; (2) they were HIV-1-infected; and (3) if frozen samples were available for resistance testing.

For each mother-child pair, we analyzed the first available HIV-1-positive sample(s) from the infant's delivery sample and the mother's. If drug resistance was detected in the newborn diagnostic sample, available follow-up samples from the infant were tested for genotypic resistance.

Other data, including the mothers' viral load values and the mothers' and infants' antiretroviral treatment histories, were obtained from the ANRS-EPF database.

### HIV-1 RNA quantification

Plasma HIV-1 RNA was quantified with the Cobas Amplicor HIV-1 Monitor 1.5 assay kit (Roche Diagnostics, Meylan, France; detection limit 400 or 40 copies/mL).

### Resistance genotyping

The ANRS consensus method was used for population-based nucleotide sequence analysis of the whole protease gene (codons 1 to 99) and codons 1 to 305 of the reverse transcriptase gene on HIV-1 RNA in plasma and HIV-1 DNA in PBMC [16]. Drug resistance mutations were identified by following the International AIDS Society-USA 2007 Drug Resistance Group guidelines [17]. Specifically, we considered the following mutations (relative to the reference wild-type (WT) strain HXB2): protease inhibitors (PI): D30N, L33F/I, M46I/L, G48V, I50L/V, V82A/F/L/S/T, I84A/C/V, and L90M; nucleoside reverse transcriptase inhibitors (NRTI): M41L, A62V, K65R, D67N, K70R, L74V, V75I, F77L, Y115F, F116Y, Q151M, M184V, L210W, T215Y/F/C/D/E/S/I/V/A/G/H/L/N and K219E/Q/R; and non nucleoside reverse transcriptase inhibitors (NNRTI): L100I, K103N, V106A/M, V108I, Y181C/I, Y188C/H/L, G190A/S, P225H, M230L, and P236L. Mixtures of WT and mutant sequences were considered drug-resistant. Interpretation of genotypic drug susceptibility was done according to the 2007 French ANRS algorithm .

### Clonal analysis of resistance in three mother-child pairs

In order to characterize the plasma and cellular viral quasispecies, clonal analyses were performed on samples from three mother-child pairs. The maternal samples were obtained at delivery and the children's samples were obtained both at birth and subsequently. These three pairs were chosen as being representative of three different situations, and because suitable plasma/cell samples for them were available. The RT or protease gene was amplified. Purified PCR products were cloned into the pCR Topo 2-1 plasmid (TOPO TA Cloning kits, Invitrogen BV, the Netherlands) as recommended by the manufacturer. DNA was purified with the Mini-Prep kit (Qiagen) and clones were analyzed by dye terminator sequencing on an ABI Prism 3100 genetic analyzer.

### Phylogenetic analysis

Mother-child clustering of *pol *sequences was confirmed by phylogenetic analysis. All sequences of HIV-1 RNA and DNA clones from each mother-child pair were aligned with Clustal W 1.7 software. Pairwise evolutionary distances were estimated with DNADist using Kimura's two-parameter method. The phylogenetic trees were then constructed with a neighbor joining method (Neighbor program implemented in the Phylip package) [18]. The reliability of each tree topology was estimated from 100 bootstrap replicates [18].

## Results

### Study population

From January 1997 to December 2004, 6170 mother-child pairs were enrolled in the ANRS-EPF cohort, representing approximately 70% of births to HIV-1-infected mothers in France. 92 newborns were infected during this period despite prophylaxis. It is important to note that the newborn samples were used to diagnose HIV infection and that the remaining stored samples were usually very limited.

HIV-1-positive plasma and/or PBMC samples from 60 children (33 boys and 27 girls) were available for drug resistance studies. Samples were also available from 32 of these children's mothers. The children's samples were obtained at a median age of 29 days (1 to 313 days), and 72% of plasma samples were collected less than 60 days after birth. The children's median HIV-1 RNA viral load at diagnosis was 4.5 log_10 _copies/ml (2.1 to 7.3 log_10_).

### Drug resistance at HIV-1 diagnosis in the infant

Twelve (20%) of the 60 newborns had resistant variants at diagnosis of HIV-1 infection, according to the 2007 IAS (International AIDS Society) list (Table 1). Six of these children were infected *in utero *and four *intrapartum*; the timing of infection could not be determined in the remaining two children as no birth sample was available. The mutations were associated with resistance to NRTI in 10 cases [thymidine analog mutations (TAMs) in six cases, T69N in one case, M184V in one case, and both mutations in two cases], NNRTI in two cases, and PI in one case.

**Table 1:** Perinatal antiretroviral exposition and drug resistance mutations in newborns and their respective mother

		**Antiretroviral perinatal exposition**					HIV-1 drug-resistance mutations*			
					
**Newborn**	**Birthyear**	**Mother**	**Intrapartum**	**Newborn**	**HIV-1 diagnosis sample**	**Viral subtype**	**in Newborns**		**in Mothers**	
								
							**HIV-1 RNA**	**HIV-1 DNA**	**HIV-1 RNA**	**HIV-1 DNA**
1	1997	ZDV	NA	ZDV	1 mo	B	70R	70R	70R/K	70R/K
2	1997	ZDV 3TC	ZDV	ZDV 3TC	birth	NA	41L, 184V	NA	41L, 184V, 215Y/F	NA
3	1997	ZDV 3TC	ZDV	ZDV 3TC	1 mo#	NA	70R, 184V	NA	70R,184V	NA
4	1998	-	-	ZDV	1 mo	CRF02	219Q/K	NA	181C	No mutation
5	1999	ZDV	-	ZDV	1 mo	B	70R	70R	NA	NA
6	1999	-	NA	ZDV	birth	F	67N/S	no mutation	no mutation	no mutation
7	2000	ZDV 3TC DDI	ZDV	ZDV	birth	CRF02	69N	69N	69N	69N
8	2001	ZDV DDI NVP	ZDV	ZDV	1 mo	B	101E, 190A	101E, 190A	NA	NA
9	2001	DDI SQV LPV/R	ZDV	ZDV	birth	B	(RT) no mutation	(RT) no mutation	(RT) 181C 210W 215D	(RT) 181C/Y 210W/L 215N/T
							(P) 10I 63P 90M	(P) 10I 63P 90M	(P) 10I 63P 90M 215Y	(P) 10I 63P 90M
10	2001	-	ZDV	ZDV	3 mo#	CRF02	103N 181C	NA	103N 181C 215Y	NA
11	2004	ZDV 3TC IDV/R	ZDV	ZDV 3TC	birth	B	184V	184V	no mutation	NA
12	2004	ZDV 3TC IDV/R	ZDV	ZDV	birth	A	70R/K	no mutation	no mutation	no mutation

* Genotypic analysis of resistance was performed on the HIV-1 diagnosis sample for the children (except for child #11, in whom resistance was analyzed at month 3) and at delivery for the mother Resistance mutations according to the IAS list 2007 were noted ((RT) reverse transcriptase; (P) protease)

ZDV = zidovudine, 3TC = lamivudine, DDI = didanosine, NVP = nevirapine, SQV = saquinavir, LPV/R = lopinavir/ritonavir, IDV/R = indinavir/ritonavir, dash "-" = untreated

# no prior available sample

NA not available

According to the 2007 ANRS algorithm, 6 of the 12 children had variants with resistance to at least one antiretroviral drug [overall frequency 10% (6/60)]. Resistance to NRTI, NNRTI and PI was observed in four children, two children and one child, respectively. One child had variants resistant to both NRTI and NNRTI (child #10, Table 1).

In all but one case, the neonates' drug resistance profiles were related to the antiretroviral drugs received by the mother and/or by the child (Table 1). Infant #10 harbored viruses with mutations associated with NNRTI resistance, without being exposed perinatally to this drug class. His mother had never received NNRTI, but she had probably been infected with NNRTI-resistant virus transmitted by her husband, who was treated with a regimen containing nevirapine, stavudine and lamivudine.

The viral subtypes were determined in 53 children, and were subtype CRF02_AG in 23 cases (43%), B in 19 cases (36%), A in 5 cases (9%) and another subtype in 6 cases (11%). Among the 10 subtyped resistant viruses, 5 (50%) belonged to subtype B, three (30%) to CRF02_AG, one to A and one to F.

DNA-based resistance results were available for 8 of the 12 children with resistant viruses in plasma. In 6 cases HIV-1 RNA and DNA harbored the same resistance mutations (Table 1), while no mutation was detected in HIV-1 DNA in the other two cases.

### Comparison of resistance mutations in the children and their mothers

Samples from 32 mother-child pairs were available, including 10 of the 12 children with resistant virus in the plasma (Table 1). The resistance pattern was the same in six mother-child pairs. In the remaining four cases the mothers harbored different mutations or no mutation. Interestingly, child #9, whose mother harbored PI resistance mutations L10I, L63P and L90M and RT resistance mutations Y181C, L210W and T215D, only harbored the PI resistance mutations. The mother was receiving didanosine, saquinavir and lopinavir/ritonavir, probably leading to the selection of a dominant PI-resistant quasispecies. Among the 22 remaining mother-child pairs, 20 mothers had wild-type viruses (in plasma), while the other two mothers harbored resistant viruses that were not transmitted to the child.

### Longitudinal resistance analysis in infected children

Longitudinal resistance studies were performed in 8 of the 12 cases in which serial samples were available (median 4 samples per child), over a median period of 52 months (Table 2). The same resistance mutations persisted in the plasma and PBMC for 6 months to 5 years, regardless of the antiretrovirals used in six children. Additional mutations had accumulated in the RNA and the DNA during failing regimens. In two children (#6, #12), no zidovudine resistance mutations were detected when zidovudine prophylaxis was discontinued. Interestingly, no resistance mutations were detected in mother samples and in birth children cells (Table 1 and 2).

**Table 2:** Longitudinal resistance analysis in newborns infected with drug resistant HIV-1

**PERSISTENCE OF RESISTANCE MUTATION**						
					**Resistance mutations in children**	
					
**Patient**	**Birth year**	**Antiretroviral regimen**	**HIV-1 diagnosis sample**	**Resistance sample (Month)**	**HIV-1 RNA**	**HIV-1 DNA**

**1**	1997	ZDV	Month 1	M1	**70R**	**70R**
		ZDV d4T ddI		M4		**70R**
		stop		M36	67N **70R **219E	
		d4T ddI EFV		M48		67N **70R **101E/K 103N/K 190S/G 219E
**7**	2000		Birth	M0	(RT) **69N**	**69N**
		d4T 3TC NFV NVP		M20	(RT) **69N **103N 181C 184V	
		3TC NVP		M26	(RT) **69N **181C 184I	
					(P) 20I 36I 71T/A 90M/L	
		stop		M50	(RT) **69N **181C	
					(P) 20I 36I	
		stop		M58	(RT) **69N **181C	
					(P) 20I 36I	
**8**	2001	ZDV	Month 1	M1	**101E 190A**	**101E 190A**
		stop		M4	**101E 190A**	
		d4T 3TC LPV/r		M12	**101E **184V **190A**	
		d4T 3TC LPV/r		M36		**101E **106I/V **190A**
		d4T 3TC LPV/r		M48	**101E **184V **190A**	**101E **106I **190A **184V
		d4T 3TC LPV/r		M55	184V **190A**	
**9**	2001	ZDV	Birth	M0	(P) **10I 63P 90M**	(P) **10I 63P 90M**
		d4T ABC NVP		M1	(P) **10I 63P 90M**	
		stop		M12		(P) **10I 63P 90M**
		ABC 3TC NFV NVP		M18	(RT) 181C 184V	
					(P) **10I 63P 90M**	
		ABC 3TC NFV NVP		M20	(RT) 181C 184V	
					(P) **10I 63P 90M**	
		d4T ABC LPV/r		M32	(RT) 181C 184V	
					(P) **10I 63P **71T **90M**	
		stop		M38	(RT) 101R/K 181C/Y	
					(P) **10I 63P **71T **90M**	
		ZDV ABC ATV/r		M48	(RT) 101R/K 215I/T	(RT) 101R/K 215I/T
					(P) **10I 63P **71T **90M**	(P) **10I 63P **71T **90M**
		ZDV ABC ATV/r		M54	(RT) 215I/T 101R/K	
					(P) **10I 63P **71T **90M**	
**10**	2001	ZDV	Month 3	M3	**103N 181C 215Y**	NA
		ZDV		M6	**103N 181C 215Y**	
		ZDV 3TC LPV/r		M24	**103N 181C **184V **215Y**	
		stop		M48	**103N 181C **184V/M **215Y/D**	**103N 181C **184V/M **215Y**
**11**	2004		Birth	M0		
		ZDV 3TC		M1		
		d4T ABC LPV/r		M3	**184V**	**184V**
		d4T ABC LPV/r		M7		**184V**
		d4T ABC LPV/r		M9	**184V**	**184V**

**REVERSION OF RESISTANCE MUTATION**						

					**Resistance mutations in children**	
					
**Patient**	**Birth year**	**Antiretroviral regimen**	**First HIV-1 positive sample**	**Resistance sampling date**	**HIV-1 RNA**	**HIV-1 DNA**

**6**	1999	ZDV	Birth	M0	**67N/S**	no mutation
		stop		M1		no mutation
		d4T ddI NFV		M12	no mutation	no mutation
**12**	2004	ZDV	Birth	M0	**70R/K**	no mutation
		ZDV		M2	**70R/K**	no mutation
		stop		M3	no mutation	no mutation
		stop		M12		no mutation
		stop		M18		no mutation
		stop		M24	no mutation	

ZDV = zidovudine, 3TC = lamivudine, ddI = didanosine, d4T = stavudine, ABC = abacavir, NVP = nevirapine, EFV = efavirenz, NFV = nelfinavir, LPV/r = lopinavir/ritonavir, ATV/r = atazanavir/ritonavir

RT: reverse transcriptase; P: protease; Persistent mutations in bold; Empty line: resistance test was not done

### Clonal and phylogenetic analysis of HIV-1 in three mother-newborn pairs

To better understand how drug-resistant HIV-1 strains detected in newborns are acquired, we conducted clonal analyses of plasma and PBMC viral populations in three mother-child pairs. The maternal samples were taken at delivery, and the children's samples were taken both at birth and at a later time.

**Figure 1:**
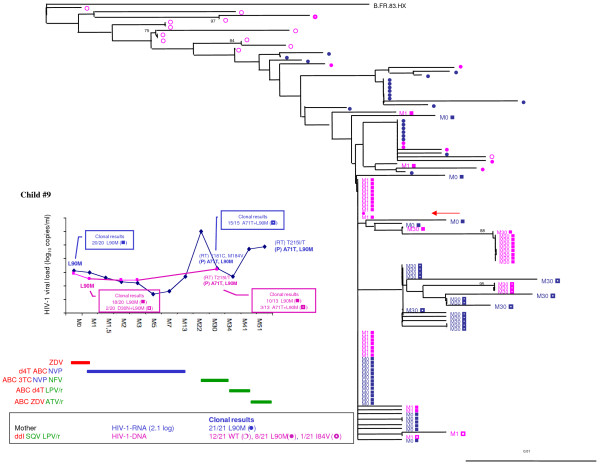
**Resistance analysis of HIV-1 RNA and DNA from the mother-child pair #9**. Time course of HIV-1 RNA and DNA levels in children with resistance mutations as detected by population-based sequencing and clonal analysis (box). Antiretroviral treatment is indicated above. Maternal antiretroviral treatment at delivery, viral RNA load, and the number of wild-type (WT) or resistant clones are indicated. In the phylogenetic tree, maternal viral clones are represented by circles and newborn viral clones by squares. M indicates the time to genotype testing in month. Wild-type quasispecies are represented by open circles and squares, and resistant quasispecies by full circles and squares. HIV-1 RNA results are in blue, and HIV-1 DNA results are in pink. The arrow indicates the maternal viral clone closest to the infant's quasispecies.

In mother-child pair #9, 110 protease gene clones were sequenced (Figure 1). In the mother, all 21 plasma clones harbored the L90M major mutation and other minor mutations. Her PBMC harbored heterogeneous variants (12/21 wild-type, 8/21 L90M and 1/21 I84V), according to the temporal archiving of resistant variants in lymphocytes during therapeutic regimens that contrasted with the homogeneity reported in the plasma under selective therapeutic pressure. In her child, who was infected *in utero*, all plasma and cellular variants harbored the L90M mutation (40/40 at birth and 28/28 at month 30), even during the period without PI selective pressure. Phylogenetic analysis confirmed the homogeneity of the child's specimens at birth with a genetic intravariability of protease gene that increased over time (from 0.003% to 0.01%). This case suggests the perinatal transmission of L90M variants with early archiving in the child's lymphocytes and persistence over time.

**Figure 2:**
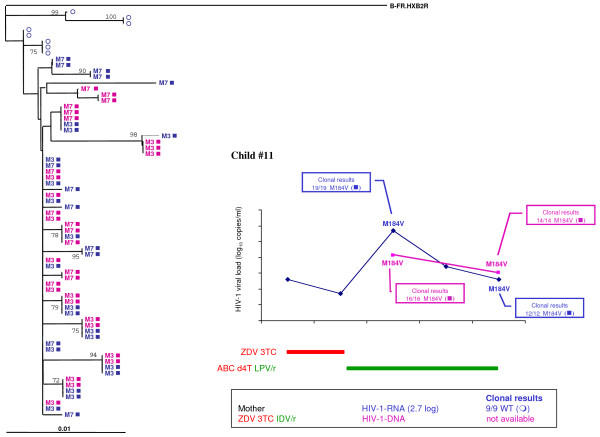
**Resistance analysis of HIV-1 RNA and DNA from the mother-child pair #11**. Time course of HIV-1 RNA and DNA levels in children with resistance mutations as detected by population-based sequencing and clonal analysis (box). Antiretroviral treatment is indicated above. Maternal antiretroviral treatment at delivery, viral RNA load, and the number of wild-type (WT) or resistant clones are indicated. In the phylogenetic tree, maternal viral clones are represented by circles and newborn viral clones by squares. M indicates the time to genotype testing in month. Wild-type quasispecies are represented by open circles and squares, and resistant quasispecies by full circles and squares. HIV-1 RNA results are in blue, and HIV-1 DNA results are in pink. The arrow indicates the maternal viral clone closest to the infant's quasispecies.

In mother-child pair #11, 70 RT gene clones were sequenced (Figure 2). The mother acquired HIV-1 infection during pregnancy and was rapidly treated with zidovudine, lamivudine and indinavir/ritonavir. The child was infected *in utero*, despite elective Cesarean section and the intensification of postnatal zidovudine prophylaxis by the addition of lamivudine. All plasma and cellular quasispecies detected in the newborn (35/35 at month 3 and 26/26 at month 7) harbored the M184V lamivudine resistance mutation. However, this mutation was not detected in the mother's delivery plasma sample (9/9 wild-type). Phylogenetic analysis confirmed low genetic intravariability (mean 0.006%) of the RT gene in the mother and her child, in keeping with the high homogeneity due to the primary infection in the child and his mother. M184V variants may have arisen during lamivudine treatment of the mother and prophylaxis of the infant, leading to the massive early lymphocyte infection and persistence of lamivudine resistance. However, we cannot exclude an abacavir-selective pressure on the M184V resistance-associated mutation or a minor maternal M184V variant transmission.

**Figure 3:**
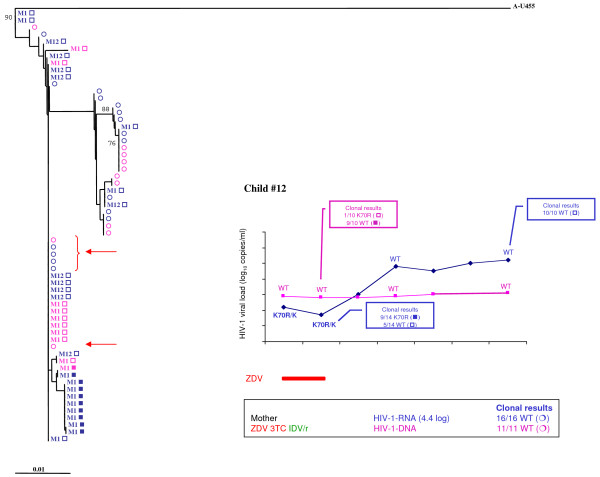
**Resistance analysis of HIV-1 RNA and DNA from the mother-child pair #12**. Time course of HIV-1 RNA and DNA levels in children with resistance mutations as detected by population-based sequencing and clonal analysis (box). Antiretroviral treatment is indicated above. Maternal antiretroviral treatment at delivery, viral RNA load, and the number of wild-type (WT) or resistant clones are indicated. In the phylogenetic tree, maternal viral clones are represented by circles and newborn viral clones by squares. M indicates the time to genotype testing in month. Wild-type quasispecies are represented by open circles and squares, and resistant quasispecies by full circles and squares. HIV-1 RNA results are in blue, and HIV-1 DNA results are in pink. The arrow indicates the maternal viral clone closest to the infant's quasispecies.

In mother-child pair #12, 61 RT gene clones were sequenced (Figure 3). The mother had advanced HIV-1 disease and poor adherence to treatment as reflected by high viral load (4.4 log_10 _copies/mL). Resistance was undetectable even by clonal analysis (28/28 wild-type). Zidovudine prophylaxis was initiated at birth and continued for 6 weeks despite the diagnosis of HIV-1 *in utero *infection in the newborn. In the child, the K70R mutation was detected in 42% of clones (10/24) at month 1 and in 0% at month 12. Genetic intravariability was low (0.005%) in the child, as expected, during primary infection. In this case, wild-type viruses were detected concomitantly in the RNA from the mother and in the DNA from the child (only 1/10 resistant clones), suggesting that most archived viruses in the child were WT viruses transmitted by the mother. Zidovudine resistance, present at the time of diagnosis, occurred during suboptimal zidovudine pressure. Zidovudine discontinuation led to the re-emergence of wild-type variants in the plasma at month 12, confirming that the reservoir consisted mainly of wild-type viruses.

## Discussion

In France, early strategies intended to prevent vertical HIV transmission are now considered suboptimal until the recommendations of HAART in 2004 [5]. Indeed, newborns are at a high risk of acquiring drug resistant variants emerging from their primary HIV-1 infection under antiretroviral selective pressure [19].

In this study, we retrospectively detected resistance mutations in 20% of children born between 1997 and 2004 who were enrolled in the ANRS-EPF cohort. Interestingly, the same frequency (7 of 34, 20%) was noted in the same cohort during the period 1994-1996 [7], even though the rate of vertical transmission was lower in the more recent period. However, whereas only zidovudine resistance was detected in 1994-1996, more varied resistance profiles were found in 1997-2004, owing to the increased diversity of antiretroviral combinations used to treat pregnant HIV-1-infected women. Resistance to NRTI remained predominant throughout the study period. The most frequent mutations were those associated with resistance to zidovudine and lamivudine, which are the only antiretroviral drugs licensed for use in neonates. Only 3% of the children (n = 2) harbored variants resistant to NNRTI, compared to 12% in American studies [13,14], probably owing to more widespread use of NNRTI-containing regimens to treat pregnant women in the USA [20]. In our study, only one child had PI resistance mutations, reflecting the recent recommendation of PI-containing regimens for PMTCT and a higher genetic barrier to resistance with ritonavir-boosted PI-containing regimens.

In most of the children studied here, the resistance profiles were related to antenatal and *post partum *antiretroviral drug exposure. This contrasts with the lack of relationship between antiretroviral drug resistance in newborns and perinatal antiretroviral exposure observed in New York State [13,14]. However, no information on maternal antiretroviral treatment and no maternal resistance genotyping were available in the latter studies.

**Figure 4:**
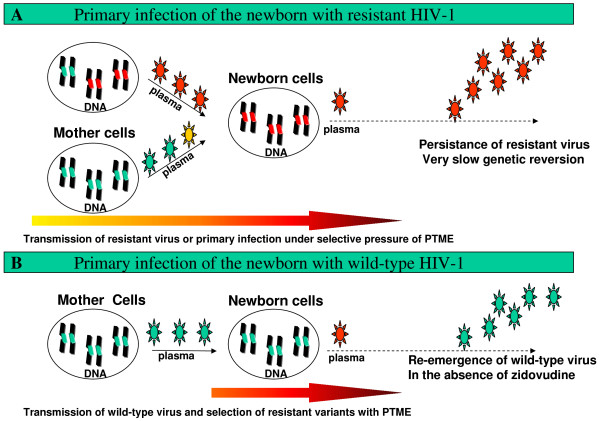
**Mechanisms of antiretroviral resistance acquisition in HIV-1-infected newborns**. Wild-type viruses are shown in green and resistant viruses in red. The length of the yellow-to-red arrow indicates the duration of perinatal prophylaxis and thus the risk of resistance selection.

The comparison of the maternal and neonatal drug resistance profiles pointed to two different mechanisms of acquisition of resistant variants by infants in the perinatal period (Figure 4). First, the infant could acquire drug-resistant variants directly from the mother (A), in one of two situations: i) the dominant variant in the mother also became dominant in the child, ii) a minor resistant variant transmitted by the mother was selected in the child during perinatal antiretroviral prophylaxis, particularly in the case of drugs such as nevirapine and lamivudine that have a low genetic barrier to resistance. Indeed, a single mutation is enough to confer high-level resistance to lamivudine or nevirapine. Moreover, selective pressure in the fetus is facilitated by the high transplacental diffusion of both these drugs [21,22]. Resistant mutations were detected early in infant lymphocytes. Clonal and longitudinal analyses showed that primary acquisition of resistant viruses was associated with long-term persistence in the infant's cellular reservoir; no matter what the subsequent treatment was.

In the second mechanism, the newborn initially acquires wild-type virus from the mother (B) (figure 4). Drug resistance can then arise during suboptimal zidovudine prophylaxis. Cloned viruses from the infants' cellular compartment were indeed wild-type, and wild-type viruses re-emerged when prophylaxis ended. Alternatively, minor resistant variants circulating in the mother may be undetectable at the clonal level in maternal samples, and/or resistant strains present in the female genital tract could be different from those circulating in the plasma [23].

Persaud *et al*. reported that drug-resistant HIV-1 in perinatally infected infants can fully populate the resting CD4+ T cell reservoir early in the course of infection and persist for years in replication-competent form [24]. Moreover, resistance acquisition and long-term persistence have been described after PMTCT with a single dose of nevirapine or lamivudine in resource-poor settings [25-27]. This long-term persistence in the cellular reservoir is reminiscent of the situation described in adults initially infected by resistant viruses [28-32]. As in adults, new resistance mutations can be acquired during suboptimal treatment with residual viral replication [31]. Our results underline the advantages of using HAART for PTMTC instead of suboptimal regimens that include drugs with a low genetic barrier to resistance and a long pharmacological half-life, as currently used in developing countries.

In the second mechanism, withdrawal of zidovudine prophylaxis led to the re-emergence of wild-type virus that had been archived during the primary infection. Once again, this resembles the situation in adults who acquire drug-resistant viruses during antiretroviral failure and in whom a dominant wild-type viral population re-emerges when antiretroviral therapy is stopped [33].

Our clonal analysis suggests that all archived viruses arising from the first mechanism are resistant (100% resistant cellular clones in children #9 and #11), compared to about 10% resistance in those arising from the second mechanism (10% resistant cellular clones in child #12).

Importantly, the main difference between primary-infection in infant and adults was the use of sub-optimal antiretroviral prophylaxies in infants that could select for resistant viruses if the infection occurs.

We observed mutations associated with resistance to at least one antiretroviral drug in six children (10%), with NRTI resistance in four, NNRTI resistance in two, and PI resistance in one. Recently, Lockman *et al*. showed that virologic failure of Triomune^® ^was more frequent in infants who were previously exposed to a single dose of nevirapine rather than a placebo [34]. In contrast, Persaud *et al*. reported that RT resistance-associated mutations did not preclude the suppression of HIV-1 replication after 24 weeks of lopinavir/ritonavir-based HAART [24]. This result together with our findings supports the use of boosted-PI regimens in children with resistance mutations or unknown resistance status.

In conclusion, our findings support resistance genotyping for children at diagnosis of HIV-1 infection, before treatment initiation, including children born to untreated mothers [35]. This approach could avoid jeopardizing drug treatment efficacy as demonstrated in adults [36]. Importantly, resistance testing in both the infant's plasma and lymphocytes would help to show whether resistance is likely to persist, with major implications for long-term treatment.

Our results also support current French recommendations to perform resistance genotyping in HIV-1-infected pregnant women in order to formulate both maternal and neonatal antiretroviral prophylaxis [5]. Finally, it is essential to use HAART and to avoid suboptimal regimens because early resistance acquisition can have drastic long-term consequences.

## Competing interests

The authors declare that they have no competing interests.

## Authors' contributions

CD, MLC carried out the resistance studies, participated in the data interpretation and drafted the manuscript. DC carried out clonage and bulk resistance analysis. VS, MB participated in the sequence alignment. SB, CD, AF, LM and RT participated in the design of the study. JW performed the statistical analysis. CR conceived of the study, and participated in its design and coordination. All authors read and approved the final manuscript.
